# 

**Published:** 1878-08

**Authors:** 


### CHICAGO MEDICAL Journal and Examiner

EDITORS:

WILLIAM H. BYFORD, A.M., M.D.,

JAS. NEVINS HYDE, A.M., M.D. FERD. C. HOTZ, M.D.

E. FLETCHER INGALS, M.D.

CHICAGO MEDICAL PRESS ASSOCIATION.

AUG. 2D 1875.

CHICAGO:

*PUBLISHED BY THE CHICAGO MEDICAL PRESS ASSOCIATION.*

188 Clark Street.

☞ Read the Notice of this Journal among Advertisements.

